# Effect of Hyperthyroidism Treatments on Heart Rate Variability: A Systematic Review and Meta-Analysis

**DOI:** 10.3390/biomedicines10081982

**Published:** 2022-08-16

**Authors:** Valentin Brusseau, Igor Tauveron, Reza Bagheri, Ukadike Chris Ugbolue, Valentin Magnon, Jean-Baptiste Bouillon-Minois, Valentin Navel, Frederic Dutheil

**Affiliations:** 1 Endocrinology Diabetology and Metabolic Diseases, CHU Clermont-Ferrand, University Hospital of Clermont-Ferrand, F-63000 Clermont-Ferrand, France; 2 Institut Génétique, Reproduction & Développement (iGReD), CNRS, INSERM, University of Clermont Auvergne, F-63000 Clermont-Ferrand, France; 3 Department of Exercise Physiology, University of Isfahan, Isfahan 81746-73441, Iran; 4 Institute for Clinical Exercise & Health Science, School of Health and Life Sciences, University of the West of Scotland, Glasgow G1 1XW, UK; 5 Department of Biomedical Engineering, University of Strathclyde, Glasgow G1 1XW, UK; 6 Physiological and Psychosocial Stress, CHU Clermont-Ferrand, University of Clermont Auvergne, F-63000 Clermont-Ferrand, France; 7 Emergency Medicine, CHU Clermont-Ferrand, University of Clermont Auvergne, F-63000 Clermont-Ferrand, France; 8 Translational Approach to Epithelial Injury and Repair, GreD, CNRS, INSERM, University of Clermont Auvergne, F-63000 Clermont-Ferrand, France; 9 Ophtalmology, CHU Clermont-Ferrand, University Hospital of Clermont-Ferrand, F-63000 Clermont-Ferrand, France; 10 Occupational and Environmental Medicine, CHU Clermont-Ferrand, University Hospital of Clermont-Ferrand, F-63000 Clermont-Ferrand, France

**Keywords:** thyroid, biomarker, autonomic nervous activity, prevention, public health, antithyroid treatment

## Abstract

The reversibility of HRV abnormalities in hyperthyroidism remains contradictory. The design of this study involves conducting a systematic review and meta-analysis on the effect of antithyroid treatments on HRV in hyperthyroidism. PubMed, Cochrane, Embase, and Google Scholar were searched until 4 April 2022. Multiple reviewers selected articles reporting HRV parameters in treated and untreated hyperthyroidism. Independent data extraction by multiple observers was stratified by degree of hyperthyroidism for each HRV parameter: RR intervals, SDNN (standard deviation of RR intervals), RMSSD (square root of the mean difference of successive RR intervals), pNN50 (percentage of RR intervals with >50 ms of variation), total power (TP), LFnu (low-frequency normalized unit) and HFnu (high-frequency), VLF (very low-frequency), and LF/HF ratio. We included 11 studies for a total of 471 treated hyperthyroid patients, 495 untreated hyperthyroid patients, and 781 healthy controls. After treatment, there was an increase in RR, SDNN, RMSSD, pNN50, TP, HFnu, and VLF and a decrease in LFnu and LF/HF ratio (*p* < 0.01). Overt hyperthyroidism showed similar results, in contrast to subclinical hyperthyroidism. Compared with controls, some HRV parameter abnormalities persist in treated hyperthyroid patients (*p* < 0.05) with lower SDNN, LFnu, and higher HFnu, without significant difference in other parameters. We showed a partial reversibility of HRV abnormalities following treatment of overt hyperthyroidism. The improvement in HRV may translate the clinical cardiovascular benefits of treatments in hyperthyroidism and may help to follow the evolution of the cardiovascular morbidity.

## 1. Introduction

Hyperthyroidism affects 0.6% people worldwide [1] with two biochemical entities, overt and subclinical hyperthyroidism [2]. It results from excessive and inappropriate production of thyroid hormones and is characterised by a hyperkinetic state [2]. One of the main complications of hyperthyroidism is cardiac arrhythmia, most often supraventricular [3]. It remains accepted that overt hyperthyroidism must be treated due to its many complications [4]. The indication to treat subclinical hyperthyroidism remains controversial. Subclinical hyperthyroidism is a risk factor for atrial fibrillation in the elderly [3] and is associated with excess cardiac mortality [5,6,7]. Cardiovascular complications of hyperthyroidism may be linked to sympathovagal imbalance [3]. Heart rate variability (HRV)—the change between two consecutive heartbeats—is a sensitive, quantitative, and noninvasive tool for detecting cardiac sympathetic and parasympathetic activity [8]. Hyperthyroidism has been associated with a reduced HRV, with increased sympathetic activity and decreased parasympathetic activity [9]. Reduced HRV is most commonly associated with a risk of arrhythmic death and is an independent predictor of cardiac morbidity and mortality [10,11,12]. Although the evaluation of antithyroid treatment on HRV parameters in hyperthyroidism has been assessed in several studies, results remain contradictory on the complete reversibility of sympathetic and parasympathetic disturbances, especially in subclinical hyperthyroidism [13,14,15,16]. Synthetic antithyroid drugs are the first-line treatment for Graves’ disease in Europe, while radioactive iodine and surgery are more popular in the US [17]. For nodular disease, radioactive iodine and surgery remain the first-line treatments [4]. Few studies have comprehensively evaluated the role of the most common variables, such as sociodemographic, clinical features or biochemical parameters of thyroid function, in the effect of antithyroid treatment on HRV parameters [18,19]. Therefore, we aimed to conduct a systematic review and meta-analysis of the impact of antithyroid treatment of overt or subclinical hyperthyroidism on HRV parameters. A secondary objective was to identify the most frequently reported explanatory variables.

## 2. Methods

The current meta-analysis was conducted in accordance with the Preferred Reporting Items for Systematic Reviews and Meta-Analyses (PRISMA) guidelines. This study did not require ethical approval as there was no human or animal experiment.

### 2.1. Literature Search

**Figure 1:**
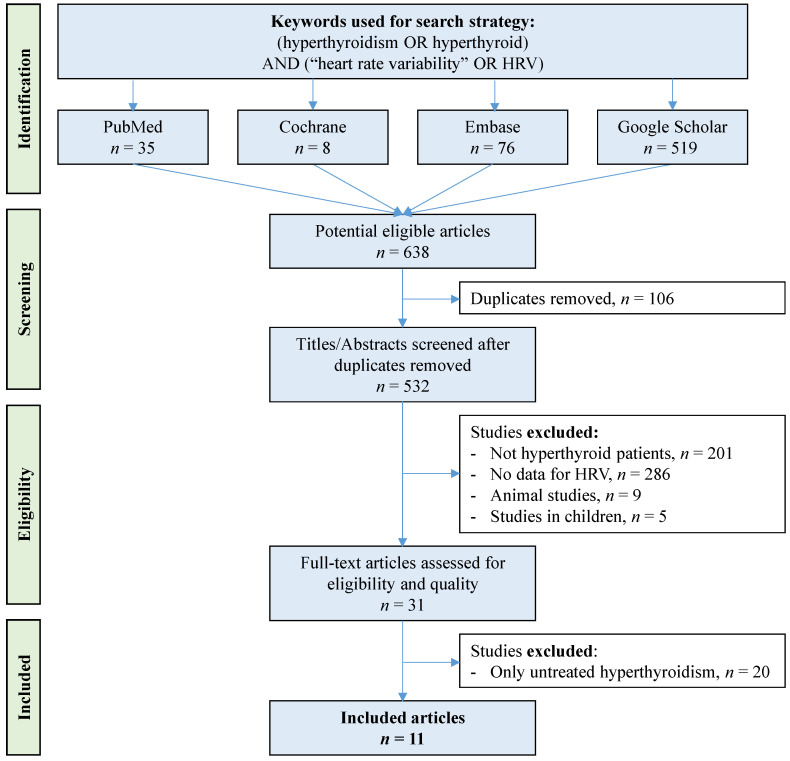
Flow chart. We followed the Preferred Reporting Items for Systematic Reviews and Meta-Analyses (PRISMA) guidelines for the search strategy. HRV: heart rate variability. The study protocol was registered and received INPALSY registration number: INPLASY202280062.

All studies that addressed the effect of hyperthyroidism treatment on HRV were reviewed. Studies were searched electronically through the major article databases (PubMed, Cochrane Library, Embase, and Google Scholar) with the following keywords: (“hyperthyroidism” OR “hyperthyroid”) AND (“heart rate variability” OR “HRV”) until 4 April 2022. To be included, studies had to describe our main primary outcome, that is, the measurement of HRV parameters in hyperthyroid patients after antithyroid therapy compared with before treatment (patients were included even if they were not their own control). Articles were included regardless of article language and years of publication, with no limitation on regional origin. Bibliographic references for all publications meeting the inclusion criteria were searched manually to identify additional studies that were not found with the electronic search. In addition, we performed ancestry searches to locate other potentially eligible primary studies in previous reviews. We excluded animal studies, studies in children, studies that evaluated the effects of other interventions in combination with antithyroid therapy, studies without frequency or time domain HRV parameters, and conferences, congresses, and seminars. Two authors (V.B. and R.B.) conducted the literature searches, reviewed the abstracts and articles independently, checked the suitability for inclusion, and extracted the data. When necessary, disagreements were solved with a third author (F.D.) (Figure 1 and File S1).

### 2.2. Data Extraction

The primary endpoint analysed was HRV parameters before and after antithyroid therapy in hyperthyroid patients. We retrieved parameters derived from the linear method of HRV measurement, which is the traditionally accepted method [8]. In the time domain, we analysed RR intervals (or normal-to-normal intervals—NN), standard deviation of RR intervals (SDNN), percentage of adjacent NN intervals varying by more than 50 milliseconds (pNN50), and root mean square of successive RR-interval differences (RMSSD). Spectral analysis [8]—also called frequency domain—is composed of three frequency ranges: low frequency (LF, 0.04 ± 0.15 Hz), high frequency (HF, 0.15 ± 0.4 Hz), and very low frequency (VLF, 0.003 ± 0.04 Hz). Power is the energy found in a frequency band [20]. The LF and HF powers are absolute powers, reported in units of ms² (square milliseconds). LFnu and HFnu are relative powers, called normalized, in the LF and HF bands, a derived index calculated by dividing LF or HF by an appropriate denominator representing the relevant total power: LFnu = LF/(LF + HF) and HFnu = HF/(LF + HF) [21]. HF power and HFnu represent parasympathetic activity [22] and are associated with RMSSD and pNN50 [20]. LF power is associated with SDNN [22] and represents both sympathetic and parasympathetic activity, but LFnu emphasizes control of sympathetic activity [8]. For example, for LF and SDNN, sympathetic [23] and parasympathetic [24] activities influence VLF [25]. We also analysed the total power (TP) and the LF/HF ratio, which is the most sensitive indicator of sympathovagal balance [8]. Secondary outcomes included clinical parameters (body mass index (BMI), blood pressure, other diseases, and treatments), hyperthyroidism characteristics (duration, aetiology, severity (i.e., overt or subclinical)), type and duration of antithyroid treatment, biological relevant parameters (free thyroxine—fT4, free triiodothyronine—fT3, thyroid-stimulating hormone—TSH), electrical parameters such as heart rate, and sociodemographic parameters (age, sex, smoking) (Table 1). All included data on HRV parameters were not under the influence of beta-blocker-type cardiac treatment.

### 2.3. Quality of Assessment

We used the Scottish Intercollegiate Guidelines Network (SIGN) criteria to check the quality of included articles with the dedicated evaluation grids. For cohort and cross-sectional studies, checklists were composed in two sections: design of the study (14 items) and overall evaluation (3 items). For clinical trials, checklists consisted of 10 items if randomized and 7 items if nonrandomized, based on the main causes of bias [26]. There were 4 possibilities of answers (yes, no, can’t say, and not applicable) (Files S2 and S3). We also used the “STrengthening the Reporting of OBservational studies in Epidemiology” (STROBE—32 items/subitems) for cohort and cross-sectional studies [27] and the Consolidated Standards of Reporting Trials (CONSORT—37 items/subitems) for randomized trials [28]. One point was assigned to each item or subitem to achieve a maximal score of 32 or 37, respectively, then converted into percentage.

### 2.4. Statistical Considerations

We used Stata software (v16, StataCorp, College Station, US) for the statistical analysis [29,30,31,32,33]. The main characteristics were synthetized for each study population and reported as mean ± standard deviation (SD) for continuous variables and number (%) for categorical variables. When data could be pooled, we conducted random effects meta-analyses (DerSimonian and Laird approach) for each HRV parameter comparing treated with untreated hyperthyroid patients [34]. A positive effect size (ES, standardised mean differences (SMD)) [35] denoted higher HRV in treated patients than in untreated. An ES is a unitless measure, centred at zero if the HRV parameter did not differ between untreated and treated patients. An ES of 0.8 reflects a large effect, that is, a large HRV increase in treated compared with untreated patients, a 0.5 moderate effect, and a 0.2 small effect. Then, we conducted meta-analyses stratified on the biochemical status of hyperthyroidism (i.e., subclinical or overt). We evaluated heterogeneity in the study results by examining forest plots, confidence intervals (CI) and I-squared (I^2^). I^2^ is the most common metric to measure heterogeneity between studies, ranging from 0% to 100%. Heterogeneity is considered low for I^2^ < 25%, modest for 25 < I^2^ < 50%, and high for I^2^ > 50%. We also searched for potential publication bias by examining funnel plots of these meta-analyses. We verified the strength of our results by conducting further meta-analyses after exclusion of studies that were not evenly distributed around the base of the funnel. When possible (sufficient sample size), metaregressions were proposed to study the relationship between each HRV parameter, clinically relevant parameters (age, sex, BMI, blood pressure), hyperthyroidism status (subclinical or overt), type and duration of treatment, and biological relevant parameters (fT3, fT4, TSH). Lastly, we repeated the aforementioned meta-analysis for each HRV parameter between treated hyperthyroid patients and healthy controls. Results were expressed as regression coefficients and 95% CI. *p*-Values less than 0.05 were considered statistically significant.

## 3. Results

An initial search produced 638 possible articles (Figure 1). The number of articles reporting the effect of antithyroid therapy on HRV in untreated hyperthyroidism was reduced to 11 articles [16,36,37,38,39,40,41,42,43,44,45] after using the selection criteria and removing duplicates. All 11 articles were written in English.

In 11 included studies, 7 were prospective [36,37,38,39,42,43,44], 2 were cross-sectional [16,41], and 2 were randomly controlled trials (RCTs) [40,45]. Included studies were published from 1996 to 2018. All included articles aimed to compare HRV treated and untreated hyperthyroid patients [16,36,37,38,39,40,41,42,43,44,45]. Sample size ranged from 18 [37] to 659 [42], for a total of 495 patients with untreated hyperthyroidism, 471 with treated hyperthyroidism, and 781 healthy controls.

Thyroid function was described clinically in all studies, but not biologically. Four articles studied HRV parameters in subclinical hyperthyroidism [16,40,43,45] and seven in overt [36,37,38,39,41,42,44]. Nine studies used antithyroid drugs [16,36,37,38,39,41,42,44,45]. Four studies used radioactive iodine treatment: three coupled with antithyroid drugs [37,42,45] and one with alone radioactive iodine treatment [43]. No study has investigated the effect of surgery. Patients achieved euthyroidism for the laboratory standards in which their thyroid workup was taken into account.

Recording of HRV measurements was an ambulatory setting with normal daily activity and during spontaneous breathing. Most studies used a 24 h Holter ECG to determine HRV [16,38,42,43,44,45]. Parameters reported were both time and frequency domains in five studies; six [16,40,41,42,43,45] reported only time domain.

More details on study characteristics (Table 2), aims and quality of articles, inclusion and exclusion criteria, characteristics of population, characteristics of hyperthyroidism, and HRV measurements and analysis are described in Supplementary Material (File S4).

### 3.1. Meta-Analysis on the Effect of Antithyroid Treatment on HRV in Hyperthyroid Patients

**Figure 2:**
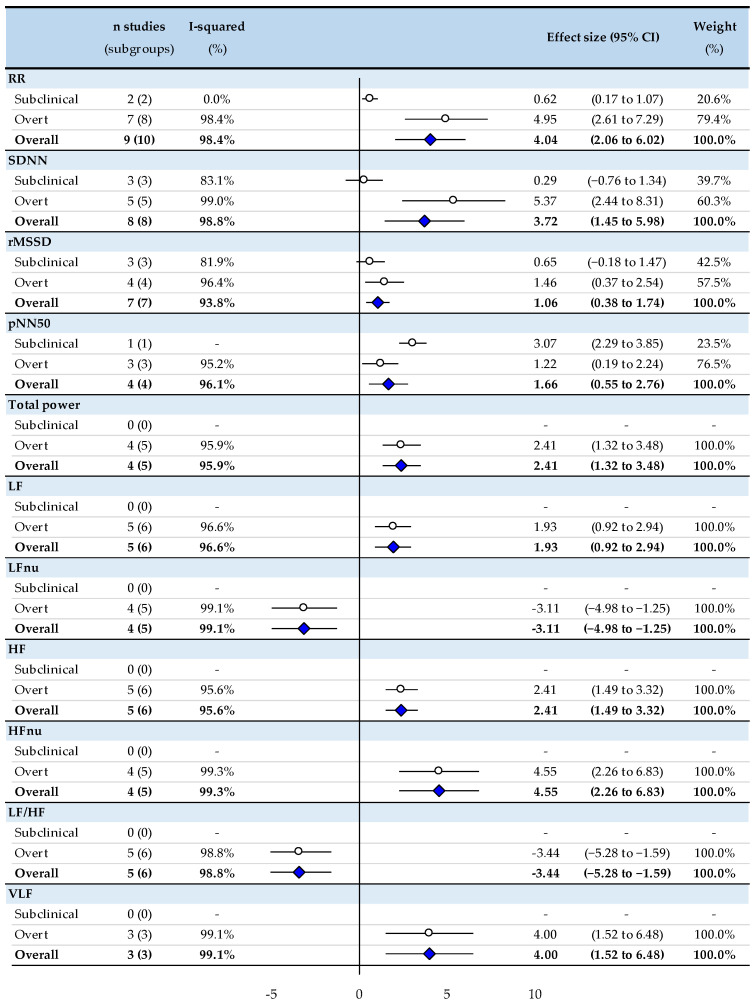
Meta-analysis of HRV parameters of untreated hyperthyroid patients compared with treated hyperthyroid patients. RR: RR intervals (or normal-to-normal intervals-NNs), SDNN: standard deviation of RR intervals, pNN50: percentage of adjacent NN intervals differing by more than 50 milliseconds, RMSSD: the square root of the mean squared difference of successive RR-intervals, LF: low frequency, LFnu: low frequency normalized—units, HF: high frequency, HFnu: high frequency—normalized units, LF/HF ratio: low frequency/high frequency ratio, VLF: very low frequency. ○: effect size stratified by subclinical or overt status; ♦: effect size for all studies.

In comparison with untreated patients, we noted strong evidence (*p* < 0.01) that treated patients had significantly higher RR intervals (ES = 4.04, 95% CI 2.06 to 6.02), SDNN (3.72, 1.45 to 5.98), RMSSD (1.06, 0.38 to 1.74), pNN50 (1.66, 0.55 to 2.76), TP (2.41, 1.32 to 3.5), LF power (1.93, 0.92 to 2.94), HF power (2.41, 1.5 to 3.32), HFnu (4.55, 2.26 to 6.83) and VLF power (4.00, 1.52 to 6.48) and lower LFnu (−3.11, −4.98 to −1.25), and LF/HF ratio (−3.44, −5.28 to −1.60) (Figure 2).

### 3.2. Meta-Analysis Stratified by Subclinical or Overt Status

In comparison with untreated patients, the following HRV parameters were increased in both overt treated hyperthyroidism and subclinical treated hyperthyroidism, respectively: RR intervals (ES = 4.95, 95% CI 2.61 to 7.29, and 0.62, 0.17 to 1.07) and pNN50 (1.22, 0.19 to 2.24, and 3.07, 2.29 to 3.85) (*p* < 0.05). Some HRV parameters were only modified in treated overt hyperthyroidism: higher SDNN (5.37, 2.44 to 8.31) and RMSSD (1.46, 0.37 to 2.54) than untreated patients (*p* < 0.05), while those parameters did not differ in subclinical hyperthyroidism. No study investigated frequency domain in subclinical hyperthyroidism. All meta-analyses had a high degree of heterogeneity (I^2^ > 90%), except for parameters explored by few studies in subclinical hyperthyroidism (RR intervals, pNN50) (Figure 2).

### 3.3. Meta-Analysis of Treated Patients Compared with Healthy Controls

**Figure 3:**
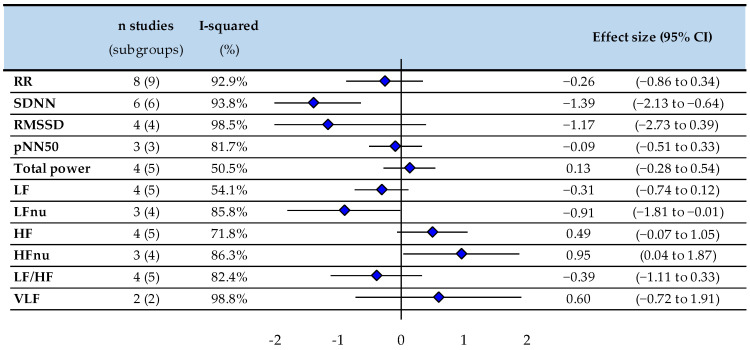
Meta-analysis of HRV parameters of treated hyperthyroid patients compared with healthy controls. RR: RR intervals (or normal-to-normal intervals-NNs), SDNN: standard deviation of RR intervals, pNN50: percentage of adjacent NN intervals differing by more than 50 milliseconds, RMSSD: the square root of the mean squared difference of successive RR-intervals, LF: low frequency, LFnu: low frequency normalized—units, HF: high frequency, HFnu: high frequency—normalized units, LF/HF ratio: low frequency/high frequency ratio, VLF: very low frequency, ♦: effect size for all studies.

Some HRV abnormalities persist in treated hyperthyroid patients (*p* < 0.05) with lower SDNN (−1.39, −2.13 to −0.64), LFnu (−0.91, −1.8 to −0.01), and higher HFnu (0.95, 0.04 to 1.87), without significant difference in other parameters (RR intervals, RMSSD, pNN50, TP, LF, HF, VLF, and LF/HF) (Figure 3). Insufficient data precluded stratification between overt and subclinical hyperthyroidism.

### 3.4. Metaregressions and Sensitivity Analyses

**Figure 4:**
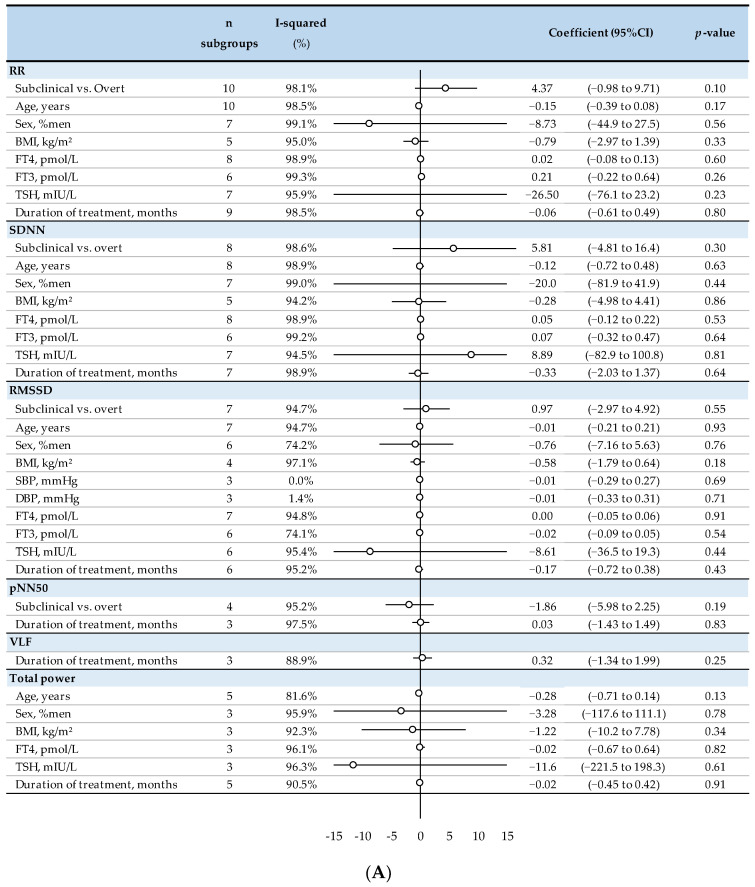

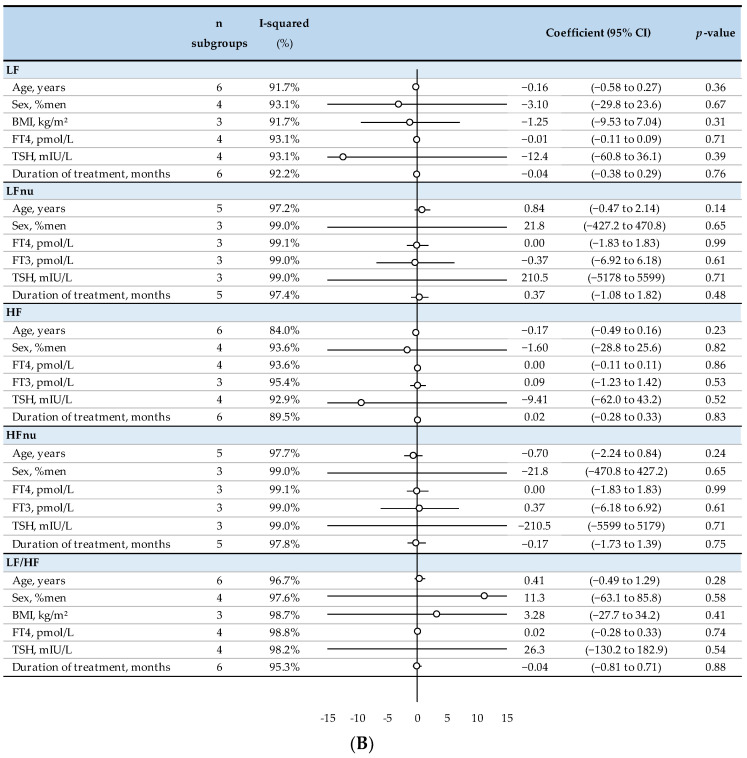
Metaregressions of factors influencing heart rate variability: RR, SDNN, RMSSD, pNN50, VLF, and total power in (**A**) and LF, LFnu, HF, HFnu, and LF/HF in (**B**) in treated hyperthyroid patients compared with untreated hyperthyroid patients. LF: low frequency, BMI: body mass index, FT4: free thyroxine, TSH: thyroid-stimulating hormone, LFnu: low frequency normalized—units, FT3: free triiodothyronine, HF: high frequency, HFnu: high frequency—normalized units, LF/HF ratio: low frequency/high frequency ratio. RR: RR intervals (or normal-to-normal intervals-NNs), BMI: body mass index, FT4: free thyroxine, FT3: free triiodothyronine, TSH: thyroid-stimulating hormone, SDNN: standard deviation of RR intervals, RMSSD: the square root of the mean squared difference of successive RR-intervals, SBP: systolic blood pressure, DBP: diastolic blood pressure, pNN50: percentage of adjacent NN intervals differing by more than 50 milliseconds, VLF: very low frequency, ○: effect size stratified by subclinical or overt status.

None of the clinical parameters (age, BMI, blood pressure, status of hyperthyroidism, duration of treatment) and biological parameters (TSH, fT4, fT3) were associated with a significant increase or decrease in time- or frequency-domain HRV parameters. The most severe patients tended to have lower RR-interval improvement following treatment compared with subclinical patients (*p* = 0.10) (Figure 4).

The meta-analyses were rerun after excluding studies that were not evenly distributed around the base of the funnel (File S5) and showed similar results, as well as following the exclusion of the study on an iatrogenic hyperthyroidism (data not shown) [40].

## 4. Discussion

The main results showed an improvement in HRV following treatments of hyperthyroidism. The decreased sympathetic and increased parasympathetic activity may have clinical and therapeutic implications.

### 4.1. Effects of Antithyroid Treatment on HRV Parameters in Hyperthyroidism

Hyperthyroidism potentiates the effect of the adrenergic system on the heart despite normal or decreased catecholamine levels [46,47,48], by an increase in the sensitivity of β-adrenergic receptors [49,50,51]. In addition, excess thyroid hormones influence parasympathetic activity by decreasing the excitability of parasympathetic nerves in the central nervous system [52] and by altering cardiac M2-muscarinic receptors [50]. When left untreated, hyperthyroidism is associated with decreased HRV with increased sympathetic activity and decreased vagal tone [9]. In general, it is accepted that effective treatment on HRV parameters should increase HF power, TP, and possibly LF power as well as relevant time domain values [20]. Antithyroid treatment allowed an improvement of the HRV parameters mainly due to a strong increase in vagal activity. The decrease in the LF/HF ratio, the greater increase in HF compared with LF, and the increase in TP are typical of increased cardiac parasympathetic activity [8,53]. However, only a partial reversibility of HRV abnormalities was noted after treatment. Indeed, we observed an improvement of HRV parameters without reaching those of healthy controls, with a persistent decrease in SDNN. SDNN is the gold standard for cardiac risk stratification among HRV parameters [8]. This suggests persistent abnormalities of cardiac autonomic function despite restoration of euthyroidism [39,40,42]. The partial reversibility of these abnormalities suggests both functional and organic parts to these disorders [40]. Indeed, irreversible changes or adaptation of the autonomic nervous system may occur with long-term exposure to excess thyroid hormones, as there are often diagnostic delays due to the specific nature of the symptoms [2]. Too short a duration of treatment may have played a role in the lack of full reversibility, which would imply that the autonomic nervous system would need more than 6 months to be restored [40]. Nevertheless, despite evidence of HRV benefits following treatment of overt hyperthyroidism, there is a lack of data in subclinical hyperthyroidism [16,40,43,45]. According to the metaregressions performed, there is no significant influence of gender, blood pressure, body mass index, duration of treatment, biochemical status of hyperthyroidism (subclinical or overt), and initial thyroid function on HRV parameters before and after antithyroid treatment in hyperthyroidism.

### 4.2. Clinical and Therapeutic Implications

The partial reversibility of HRV abnormalities in hyperthyroidism after treatment has clinical and therapeutic implications. The cardiovascular system is one of the main targets of thyroid hormone action [54]. Indeed, hyperthyroidism is associated with several diseases, such as coronary heart disease [5], heart failure [55], cardiovascular mortality [56], and an increased risk of atrial fibrillation [57]. Low TSH level is the main risk factor for the development of atrial fibrillation [58,59] with a more than fivefold probability [60,61] due to a direct action of thyroid hormones and to an effect mediated by sympathovagal imbalance [62]. Increased β-adrenergic receptor sensitivity in the atria and vagal reduction have been observed before the onset of paroxysmal atrial fibrillation [63,64]. The importance of sympathovagal balance in the pathophysiology of atrial fibrillation indicates that antithyroid therapy may have an impact on its prevention with a significant decrease in morbidity and mortality from embolic events [60,65]. Indeed, increased parasympathetic activity and decreased sympathetic stimulation prevent arrhythmia in experimental and clinical models [66,67]. HRV is associated with cardiovascular risk in many conditions, mainly heart failure, myocardial infarction, and diabetic autonomic neuropathy [8,68]. Decreased HRV has been shown to predict an increased risk of sudden cardiac death [10] and total cardiac mortality [11]; decreased LF power was a strong predictor of sudden death independent of other variables [69], and decreased VLF was associated with the risk of arrhythmic mortality [70]. The reversibility of these anomalies after treatment suggests that antithyroid therapy decreases the risk of arrhythmia and reflects a health benefit in the hyperthyroid patient [23]. This increase in HRV also indicates a better adaptation to microenvironmental changes in these patients with an adaptable and dynamic autonomic nervous system [71]. We might ask whether symptomatic treatment with a β-blocker would not be sufficient for the reversibility of these abnormalities. One study showed that propranolol had an effect on heart rate with no change in HRV parameters, whereas it is one of the most effective treatments [72]. There are also nondrug methods, such as slow breathing exercises to improve vagal tone, but these have not been studied in hyperthyroidism [73]. This suggests that antithyroid treatment remains the only option for reversing sympathovagal imbalance.

### 4.3. Limitations

Theoretically, individual studies are subject to publication bias and have their own limitations, so all meta-analyses have limitations [74]. The use of broader keywords in the search strategy limits the number of missing studies, although the meta-analysis was based on a moderate number of studies [75]. Despite the rigorous inclusion criteria for studies within the meta-analysis, the quality of the studies varied [38,42,43,44]. Only two studies were RCT [40,45], precluding robust conclusions for our meta-analyses [75]. The inclusion criteria, exclusion criteria, and data from each included study were similar but not identical; this may have affected our results [76]. In addition, all studies were monocentric, limiting the generalizability of our results [76]. The variables extracted from the declarative data of each included study were also a putative bias [74]. We limited the influence of extreme results and heterogeneity by exclusion of outliers [77,78]. Studies also differed in measurement conditions, such as in the duration of recording of HRV parameters [37,38]. We did not compute meta-analysis on the nonlinear assessment of HRV, but it has been poorly studied in treated hyperthyroidism and is controversial—its results being nonproportional, maximizing minimal or major changes [79,80]. The aetiology and duration of hyperthyroidism before treatment were poorly reported, precluding further analysis. We were unable to perform metaregressions on the type of treatment of hyperthyroidism because most studies used different molecules [16,36,37,38,39,41,42,43,44,45] and treatments were often combined [37,42,45] (i.e., synthetic antithyroid drugs associated with radioactive iodine). Similarly, the lack of data on the spectral analysis of subclinical hyperthyroidism was inconclusive on the reversibility of HRV abnormalities. The management of subclinical hyperthyroidism is not based on any good-quality randomized clinical trials, but only on expert or learned society recommendations with a low level of evidence [81,82]. The majority of subclinical hyperthyroidism does not progress to overt hyperthyroidism [81], but there is still a risk factor for arrhythmia by atrial fibrillation, and it is associated with excess cardiac mortality [3]. Effective treatment of HRV abnormalities may reduce cardiovascular complications in subclinical hyperthyroidism [5,8].

## 5. Conclusions

We showed a partial reversibility of HRV abnormalities following treatment of overt hyperthyroidism. No conclusions could be made about the effect of treatment in subclinical hyperthyroidism because of a lack of data. The improvement in HRV may translate the clinical cardiovascular benefits of treatments in hyperthyroidism and may help to follow the evolution of the cardiovascular morbidity. Further studies should also focus on subclinical hyperthyroidism and on the putative benefits of early treatments in the prevention of cardiovascular complications.

## Figures and Tables

**Table 1:** Descriptive characteristics of HRV parameters.

HRV Parameters		
Acronym (Unit)	Full Name	Signification
Time domain		
RR (ms)	RR-intervals (or normal-to-normal intervals—NN) (i.e., beat-by-beat variations of heart rate)	Overall autonomic activity
SDNN (ms)	Standard deviation of RR intervals	Correlated with LF power
RMSSD (ms)	Root mean square of successive RR-interval differences	Associated with HF power and hence parasympathetic activity
pNN50 (%)	Percentage of adjacent NN intervals varying by more than 50 milliseconds	Associated with HF power and hence parasympathetic activity
**Frequency domain**		
TP (ms^2^)	Total power i.e., power of all spectral bands	Overall autonomic activity
VLF (ms^2^)	Very low frequency (0.003 to 0.04 Hz)	Thermoregulation, renin-angiotensin system
LF (ms^2^)	Power of the high-frequency band (0.04–0.15 Hz)	Index of both sympathetic and parasympathetic activity, with a predominance of sympathetic
HF (ms^2^)	Power of the high-frequency band (0.15–0.4 Hz)	Represents the most efferent vagal (parasympathetic) activity to the sinus node
LF/HF	LF/HF ratio	Sympathovagal balance

**Table 2:** Characteristics of included studies.

Study	Country	Design	Subgroup	Intervention	Duration *	Healthy Controls	Age, Years	Sex (% men)	Before Treatment			After Treatment			ECG, min	HRV Parameters
									*n*	fT4, pmol/L	TSH, mIU/L	*n*	fT4, pmol/L	TSH, mIU/L		
Burggraaf 2001	The Netherlands	Prospective	1 group: Overt	Obtaining 1 month of euthyroidism after antithyroid treatment (ATD **, thiamazole)	5 ± 3.5	Yes	38.9 ± 9.7	7.1%	14	64.8 ± 18.9	0.2 ± 0.3	14	16.5 ± 3.6	1.4 ± 1.9	20	RR, SDNN, TP, LF, HF, LF/HF
Cacciatori 1996	Italy	Prospective	1 group: Overt	Treatment by ATD (methimazole) for 12–18 months	15 ± 3	Yes	-	-	8	38.0 ± 4.0	<0.01	8	10.4 ± 1.4	1.8 ± 0.6	10	RR, TP, LF, HF, LF/HF
Cai 2018	China	Prospective	1 group: Overt	Antithyroid treatment by ATD (carbimazole, PTU ***) ± RT ****	3.7 ± 0.7	Yes	35.0 ± 13.0	38.6%	57	122 ± 104	<0.01	50	21.9 ± 7.7	4.0 ± 2.1	1440	RR, SDNN, RMSSD, pNN50, LF, HF, VLF, LF/HF
Chen 2006	Taiwan	Prospective	1 group: Overt	Antithyroid treatment by ATD	6.0 ± 3.0	Yes	31.0 ± 2.0	9.4%	32	72.9 ± 3.1	<0.01	28	15.2 ± 1.0	1.9 ± 0.5	30	RR, TP, LF, HF, VLF, LF/HF
Eustatia-Rutten 2008	The Netherlands	Randomised trial	1 group: Subclinical	Stop TSH-suppression treatment with restauration of euthyroidism at 6 months	6.0 ± 0.0	Yes	51.0 ± 10.5	33.3%	12	22.6 ± 3.9	0.1 ± 0.1	12	18.5 ± 4.1	3.0 ± 2.3	16	RR, SDNN
Falcone 2014	Italy	Cross-sectional	2 groups:	No intervention—Two different groups		Yes									1440	RR, SDNN, RMSSD, pNN50
			Untreated subclinical		-		67.0 ± 14.1	17.9%	28	15.4 ± 7.3	0.2 ± 0.1	-	-	-		
			Treated subclinical by ATD (tapazole)		Unspecified		66.3 ± 11.0	35%	-	-	-	20	14.5 ± 2.8	1.7 ± 0.7		
Kabir 2009	Bangladesh	Cross-sectional	2 groups:	No intervention—Two different groups (untreated vs treated)		Yes									5	RR, SDNN, RMSSD
			Untreated overt		-		38.9 ± 2.4	-	30	51.4 ± 7.6	0.02 ± 0.01	-	-	-		
			Treated overt by ATD		2.0 ± 0.5		40.8 ± 4.8	-	-	-	-	30	30.4 ± 4.2	0.02 ± 0.0		
Kaminski 2012	Poland	Prospective	1 group: Subclinical	Obtaining 6 months of euthyroidism after antithyroid treatment (RT)	6.0 ± 0.0	No	45.9 ± 11.0	15.9%	44	14.2 ± 2.4	0.2 ± 0.1	44	13.1 ± 1.8	1.3 ± 0.8	1440	RMSSD
Osman 2004	United Kingdom	Prospective	1 group: Overt	Antithyroid treatment by ATD or RT	6.4 ± 1.2	Yes	49.0 ± 12.5	23.2%	224	35.8 ± 12.0	-	219	12.8 ± 2.0	-	1440	RR, SDNN, RMSSD, pNN50
Wustmann 2008	Switzerland	Prospective	1 group: Overt	Antithyroid treatment by ATD (carbimazole, PTU)	16.0 ± 6.0	No	43.0 ± 11.0	10.7%	28	27.1 ± 14.1	<0.01	28	13.1 ± 3.9	2.2 ± 1.6	1440	RR, SDNN, RMSSD, pNN50, LF, HF, VLF, LF/HF
Yönem 2002	Turkey	Randomised controls trial	1 group: Subclinical	Antithyroid treatment by ATD (PTU) and RT	6.0 ± 0.9	Yes	38.7 ± 1.4	10%	10	16.4 ± 0.1	0.2 ± 0.03	10	12.7 ± 1.4	0.8 ± 0.2	1440	SDNN, RMSSD

* Duration: Duration of treatment, months; ** ATD: Antithyroid drugs; *** PTU: Propylthiouracil; **** RT: Radioiodine treatment. fT4: free thyroxine, TSH: thyroid-stimulating hormone, RR: RR intervals (or normal-to-normal intervals-NNs), SDNN: standard deviation of RR intervals, pNN50: percentage of adjacent NN intervals differing by more than 50 milliseconds, RMSSD: the square root of the mean squared difference of successive RR-intervals, TP: total power, LF: low frequency, HF: high frequency, VLF: very low frequency, LF/HF ratio: low frequency/high frequency ratio. -: no data.

## Data Availability

All relevant data were included in the paper.

## Supplementary Materials

The following supporting information can be downloaded at: https://www.mdpi.com/article/10.3390/biomedicines10081982/s1, File S1. Details for the search strategy used within each database. File S2. Methodological quality of included studies using the SIGN checklist. File S3. Quality of included studies. Methodological quality of included studies using the SIGN checklist. Methodological quality of included studies using the SIGN checklist, by study. SIGN checklist for cohort studies. SIGN checklist for controlled trial studies. Methodological quality of included studies using STROBE and CONSORT checklists, by study. File S4. Detailed meta-analysis in treated hyperthyroid patients compared with untreated for each HRV parameter: RR intervals, SDNN, RMSSD, pNN50, TP, LF, HF, VLF, and LF/HF. File S5. Metafunnels.
