# Supplementary material for: Effect of Hyperthyroidism Treatments on Heart Rate Variability: A Systematic Review and Meta-Analysis
Source: Biomedicines. 2022 Aug 16;10(8):1982. doi: 10.3390/biomedicines10081982 (PMC9405700; doi:10.3390/biomedicines10081982)

Figure S5. Detailed meta-analysis in treated hyperthyroid patients compared with untreated for each HRV parameters: RR intervals, SDNN, RMSSD, pNN50, TP, LF, HF, VLF, LF/HF

RR: RR intervals (or normal-to-normal intervals-NNs), SMD: standardised mean differences (effect size)

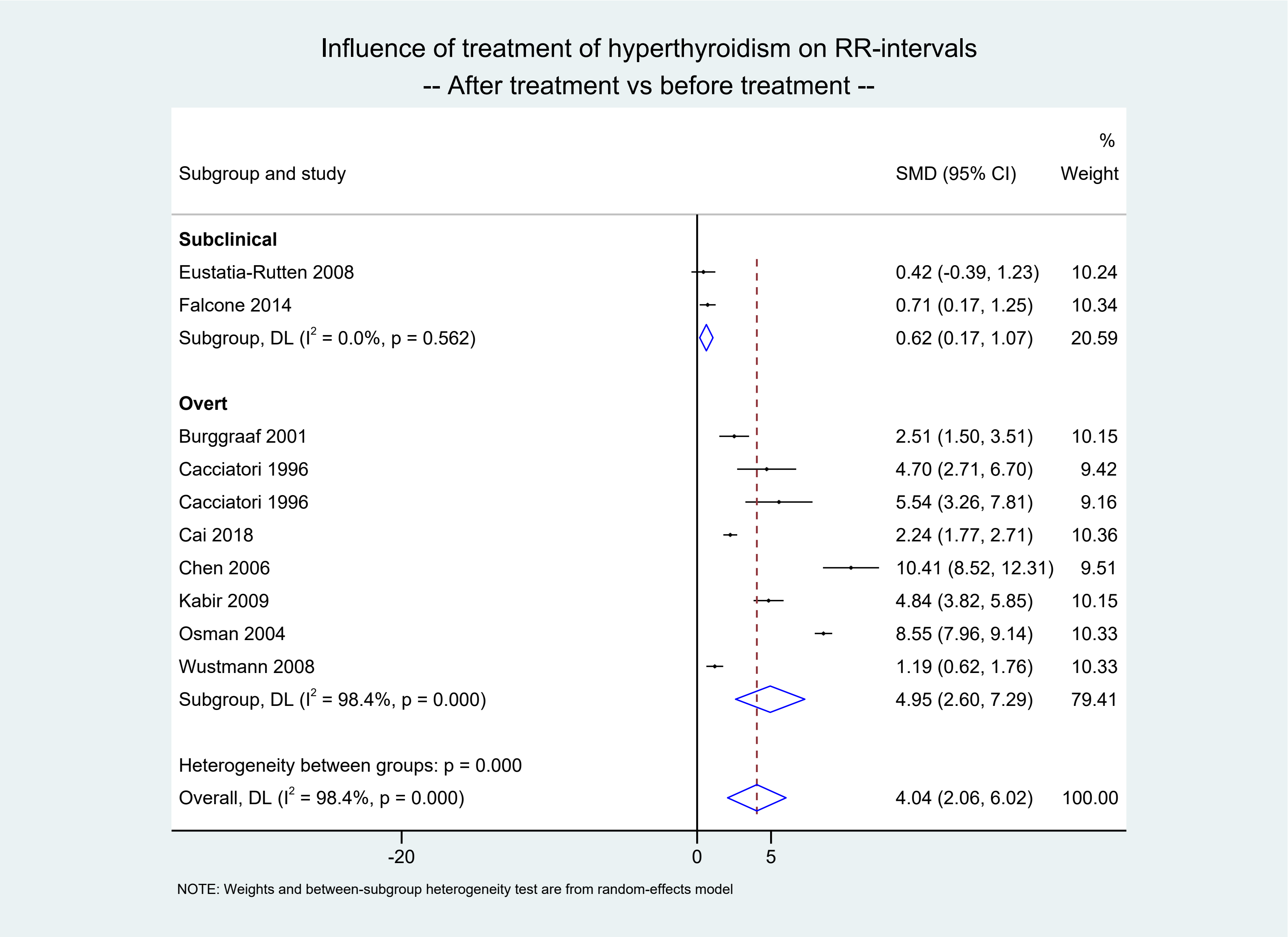

SDNN: standard deviation of RR intervals; SMD: standardised mean differences (effect size)

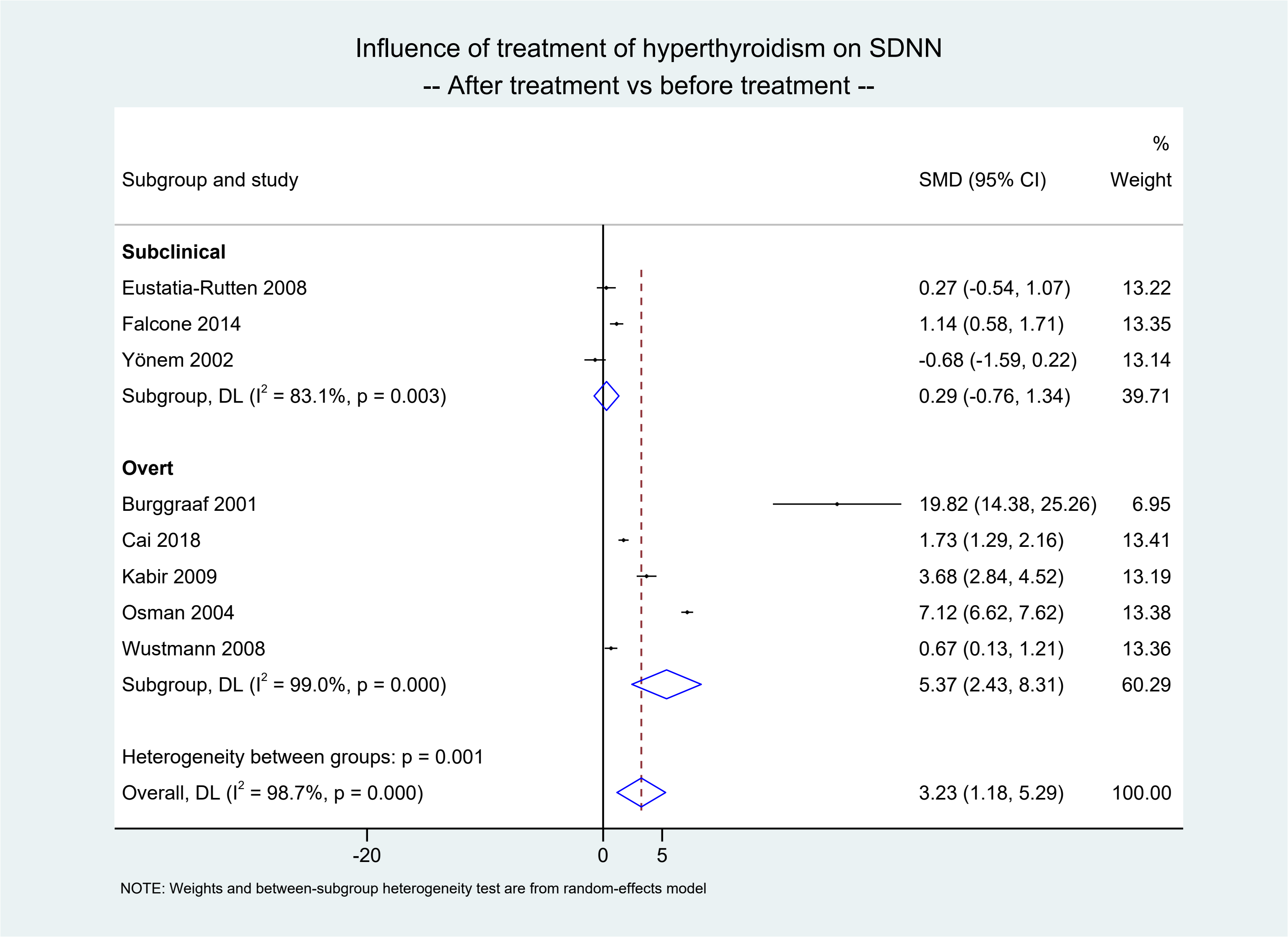

RMSSD: the square root of the mean squared difference of successive RR-intervals; SMD: standardised mean differences (effect size)

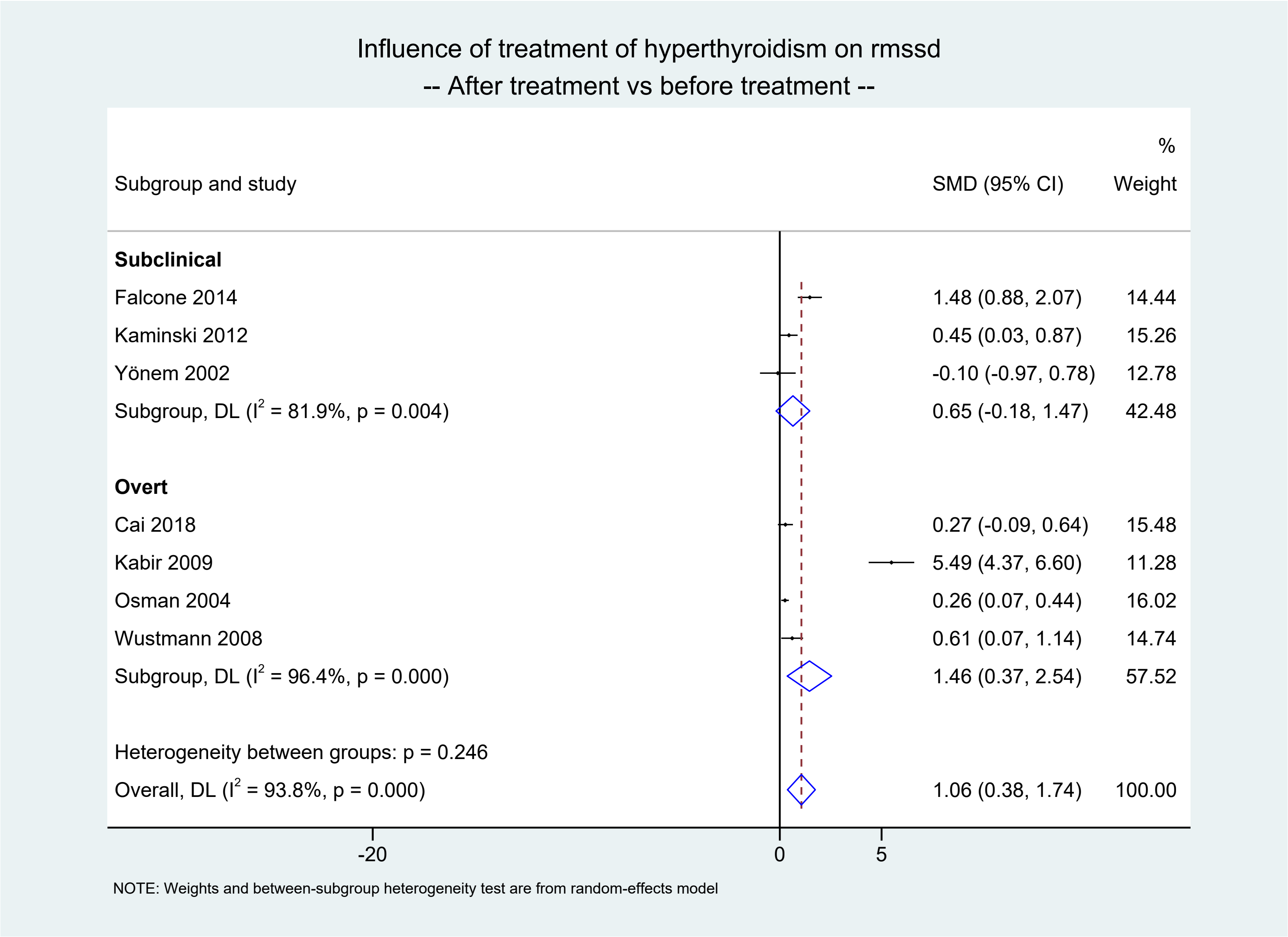

pNN50: percentage of adjacent NN intervals differing by more than 50 milliseconds, SMD: standardised mean differences (effect size)

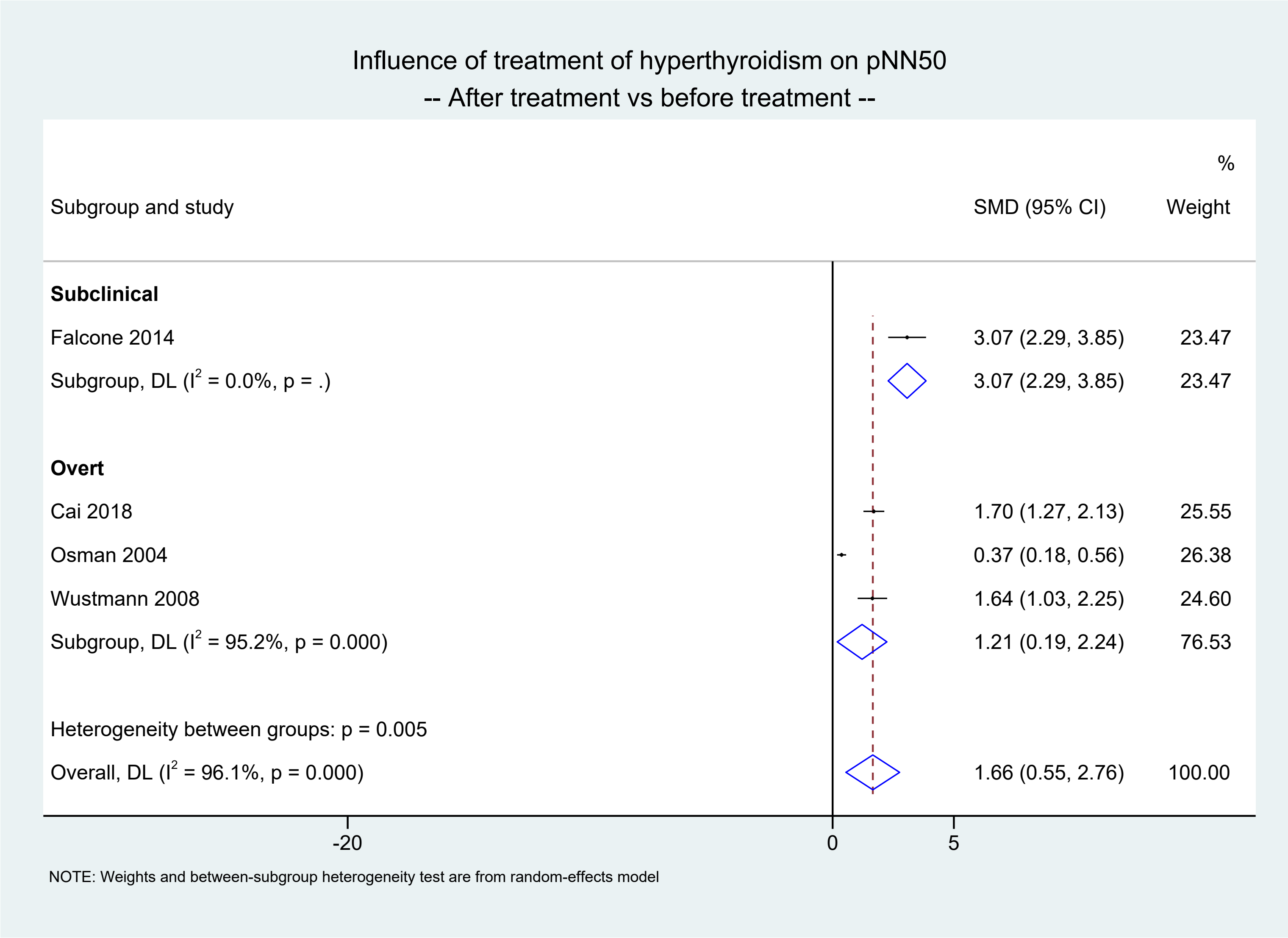

SMD: standardised mean differences (effect size)

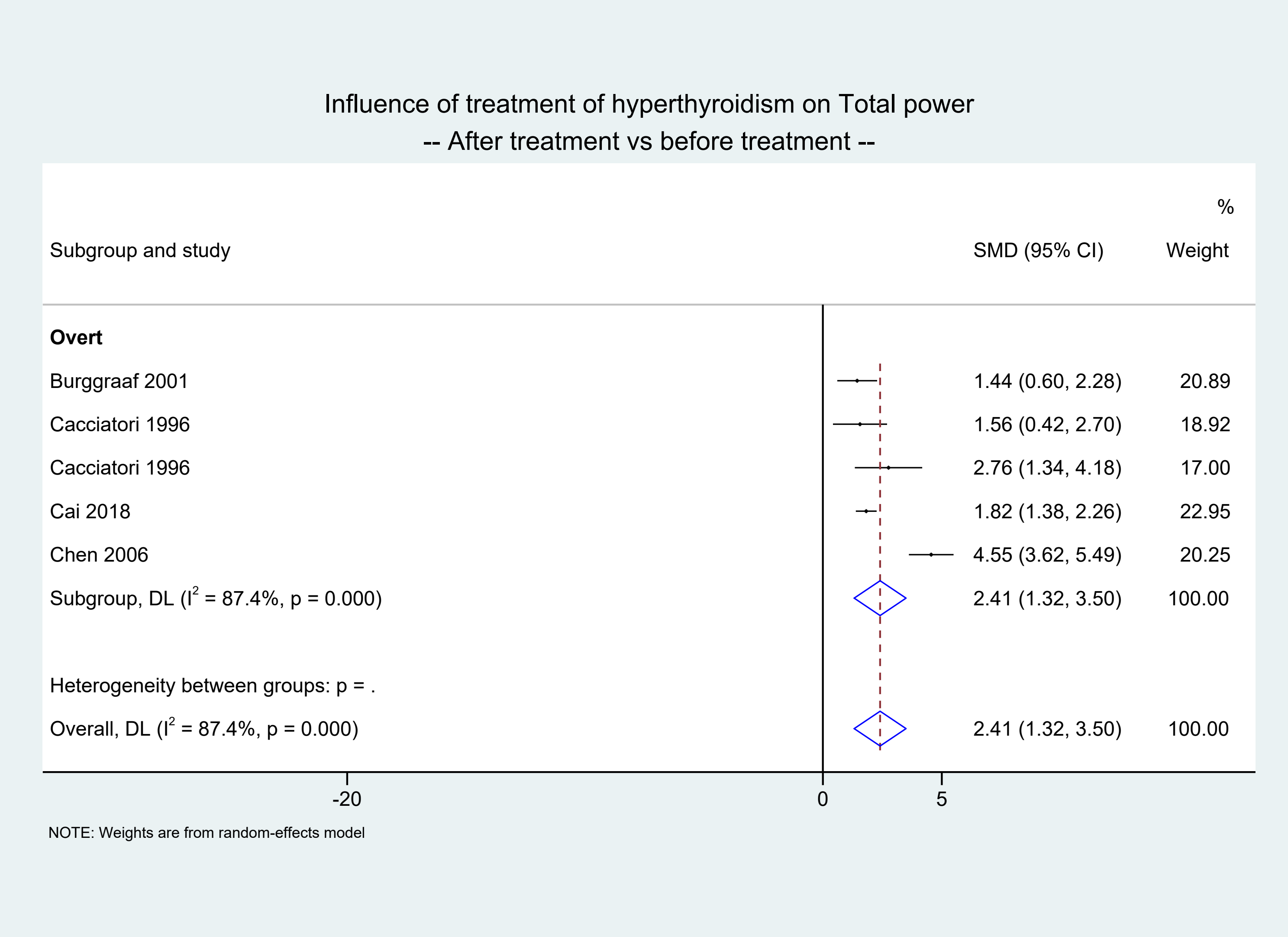

LF: low frequency; LFnu: low frequency normalized – units, SMD: standardised mean differences (effect size)

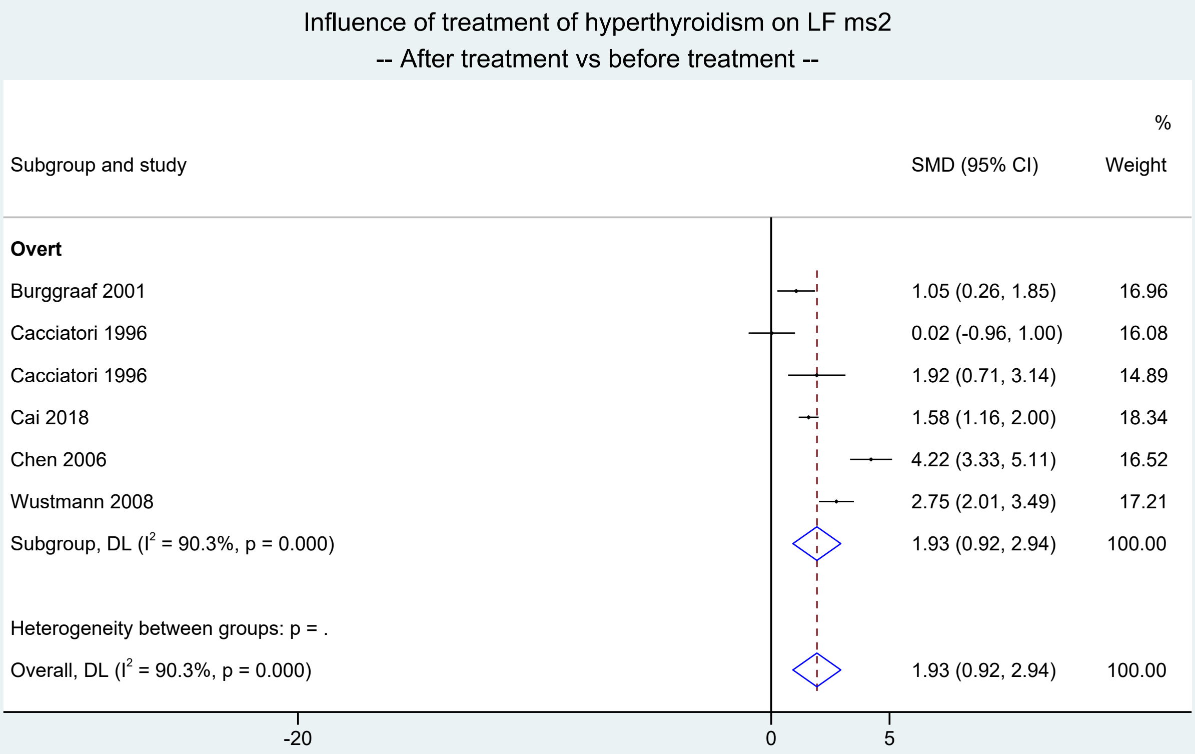

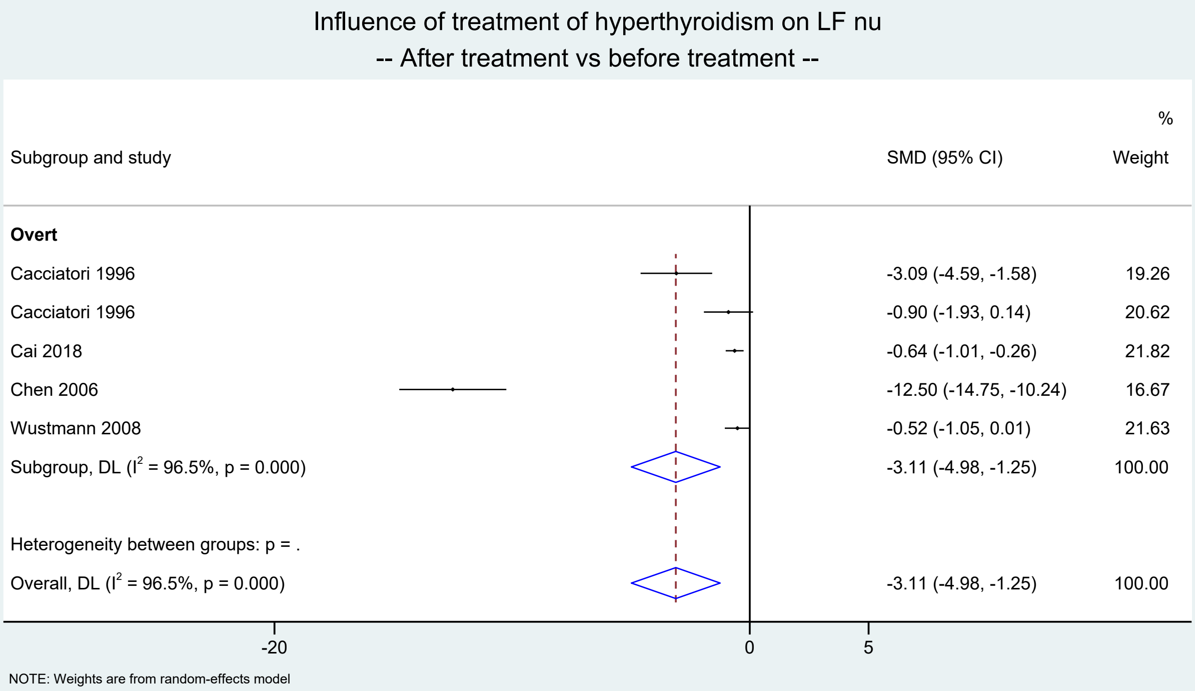

HF: high frequency; HFnu: high frequency – normalized units; SMD: standardised mean differences(effect size)

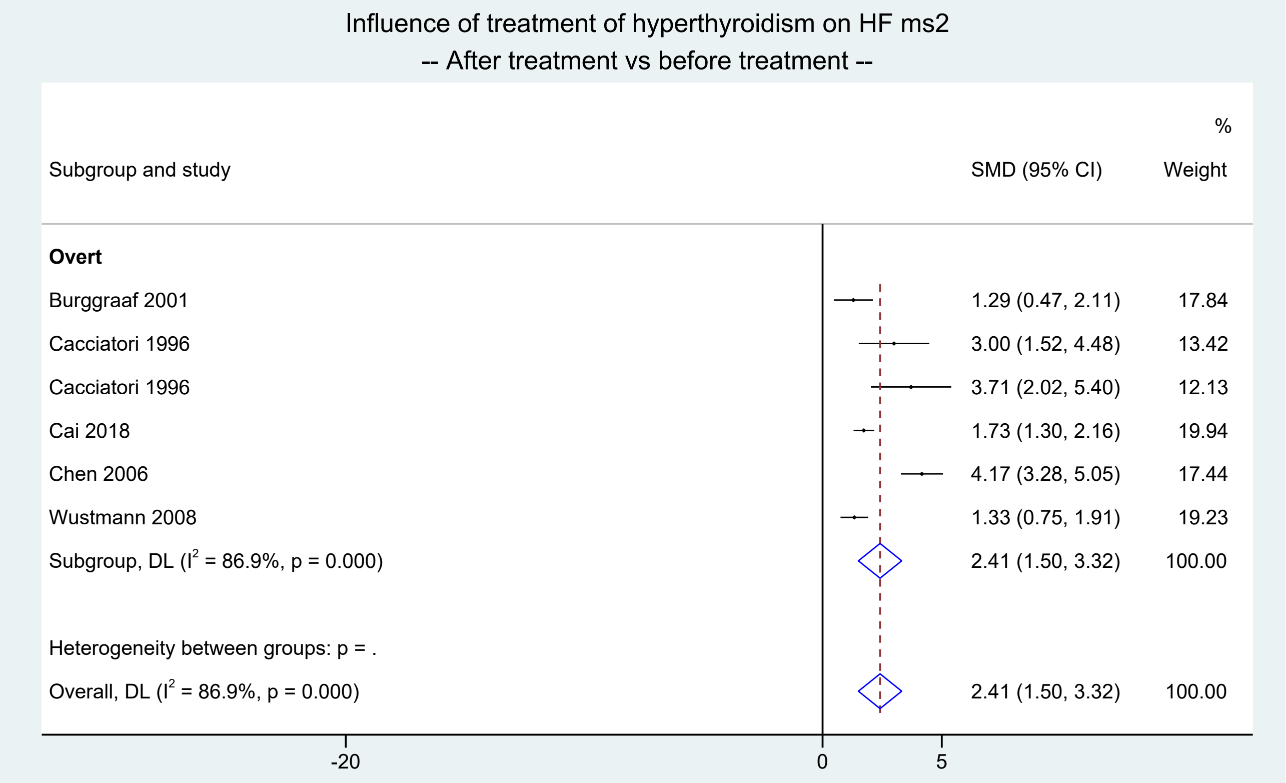

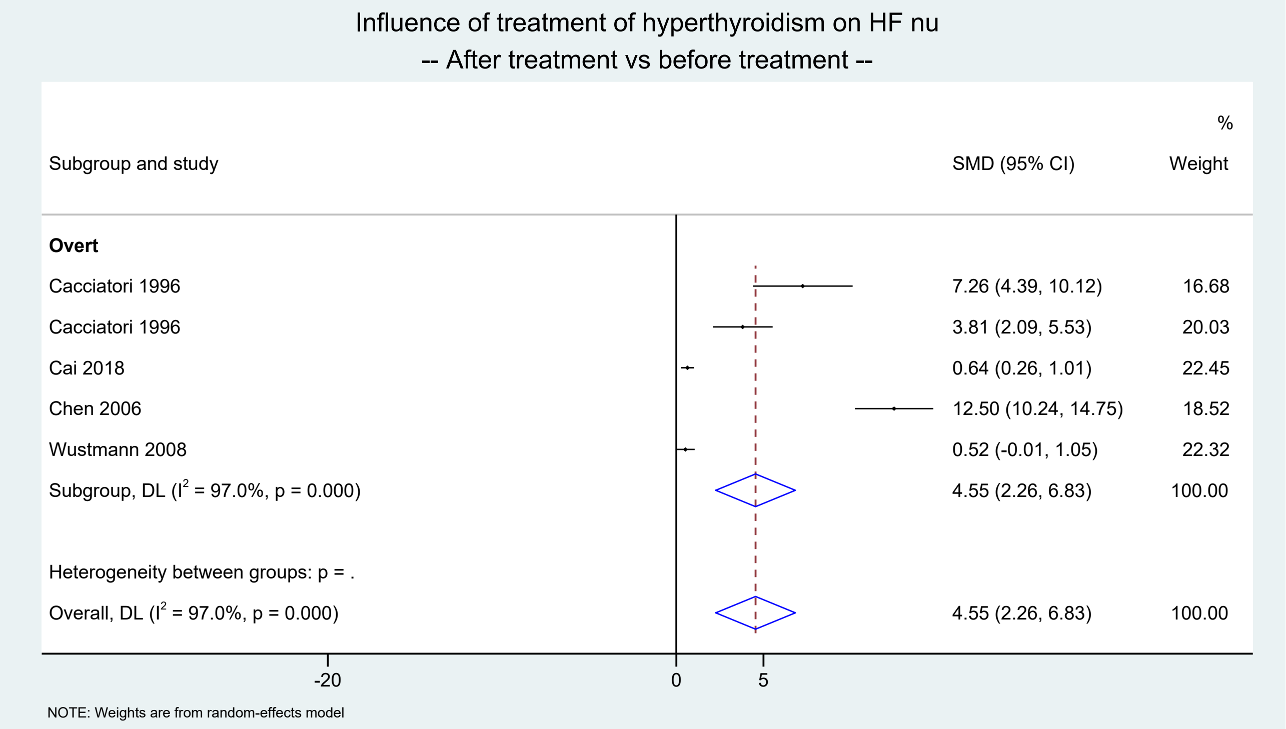

VLF: very low frequency; SMD: standardised mean differences (effect size)

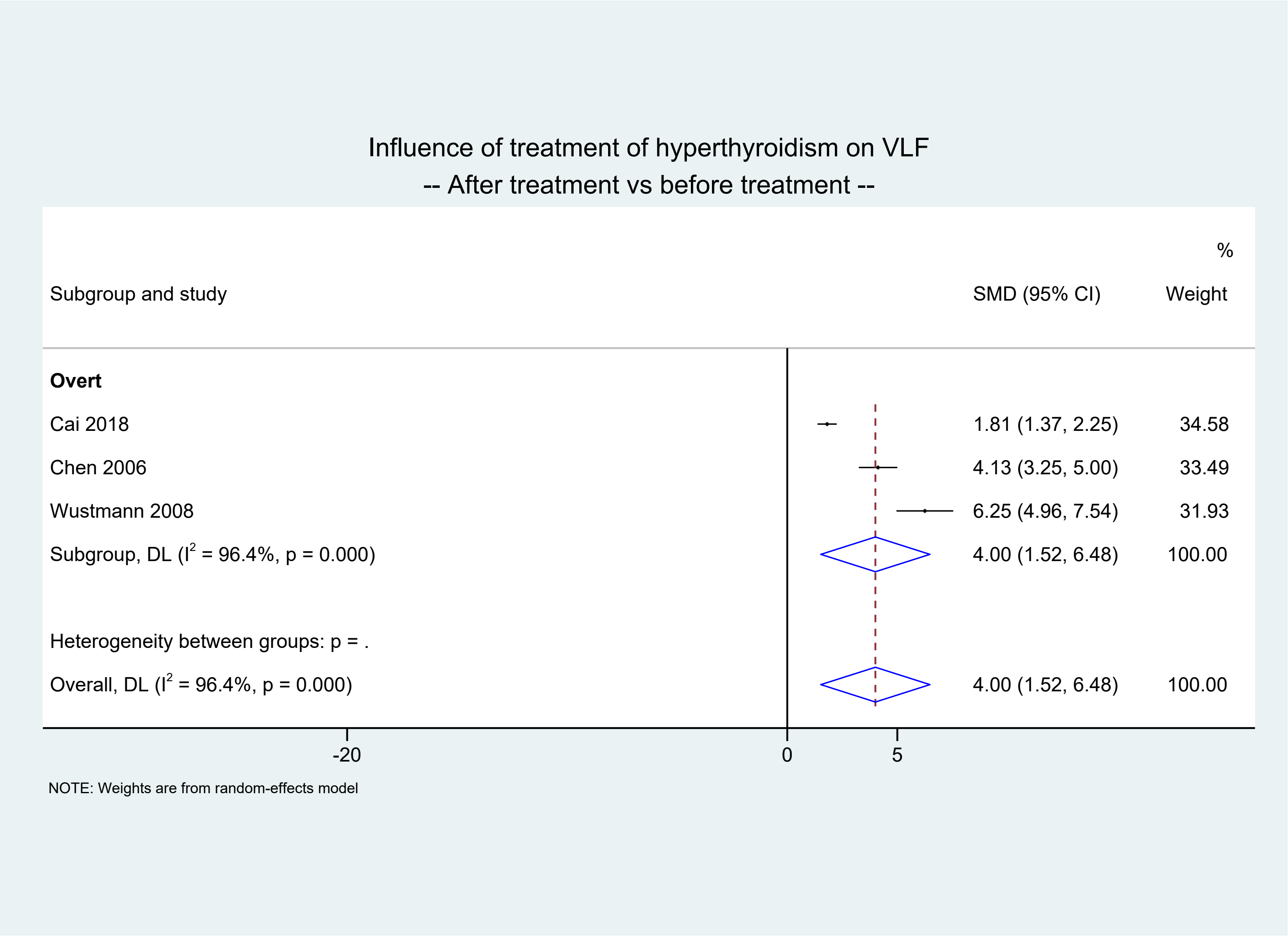

LF/HF ratio: low frequency / high frequency ratio; SMD: standardised mean differences (effect size)

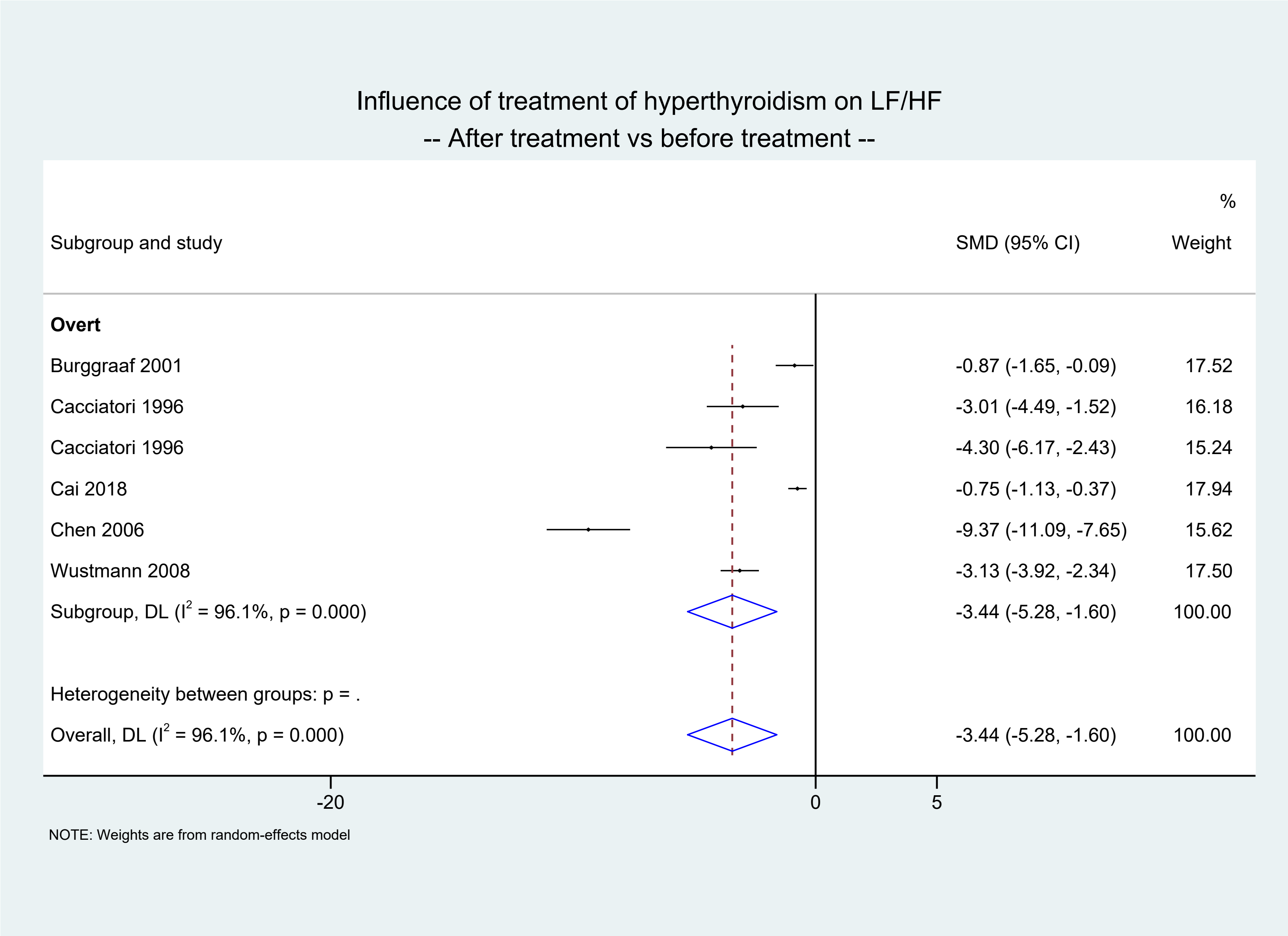

Supplement: Supplementary file 1 [file biomedicines-10-01982-s001.zip › BrusseauAnnexeSupplementaryFive.pdf]
