# Supplementary material for: Effect of Hyperthyroidism Treatments on Heart Rate Variability: A Systematic Review and Meta-Analysis
Source: Biomedicines. 2022 Aug 16;10(8):1982. doi: 10.3390/biomedicines10081982 (PMC9405700; doi:10.3390/biomedicines10081982)
Supplement: Supplementary file 1 [file biomedicines-10-01982-s001.zip › BrusseauAnnexeSupplementaryFour.pdf]

Figure S4. Study designs, Aims of included articles, Quality of articles, Inclusion and exclusion criteria of included studies, Characteristics of population, Characteristics of hyperthyroidism, and HRV measurements and analysis.

Study designs

In 11 included studies, seven were prospective [36–39,42–44], two were cross-sectional [16,41], and two were randomly controlled trials (RCT) [40,45]. Included studies were published from 1996 to 2018 and conducted across two continents (Europe – 7 studies, Asia – 4 studies).

Quality of articles

Using SIGN, mean quality score was 60.4 ± 7.0% for cohort studies, ranging from 50.0% [43,44] to 68.8% [38,42] for Yes responses, and 70.0 ± 10.0% for RCT, ranging from 60% [45] to 80% [40]. Few studies were at high level of proof mainly due to the lack of visibility on exposure status. Scores were 60.7 ± 5.8% for observational studies using STROBE, ranging from 51.5% [36] to 69.7% [38] and 57.0 ± 4.2% for RCT using CONSORT, ranging from 54.0% [45] to 60.0% [40]. Overall, the studies scored worst in the methods section (Figure S2 and S3).

Aims of included articles

All included articles aimed to compare HRV treated and untreated hyperthyroid patients. Two studies were cross-sectional [16,41]. Seven had two measures within the same patients (before and after treatment) [36–39,42–44], and five of them also included healthy controls [36–39,42]. Two RCT studied untreated hyperthyroid patients, separating them into two groups, one treated and one untreated [40,45]. Three studies measured HRV after restoration of euthyroidism [36,40,43]. Others studies [16,37–39,41,42,44,45] measured HRV at the end of a defined period of treatment, even if euthyroidism was not completely reached.

Inclusion and exclusion criteria of included studies

Most studies included patients with newly diagnosed hyperthyroidism before treatment [36–39,41,44], except three that included patients with untreated hyperthyroidism over 6 months [45], 12 months [43], or 10 years [40]. Other studies did not indicate duration of hyperthyroidism [16,42]. Most studies included patients regardless of their age, sex, or BMI, except few studies that included only patients between 30-50 years [41] or with a BMI between 17.3-23kg/ms^2 [37]. In all studies, patients were not required to have antithyroid treatment at inclusion, except two cross-sectional studies that compared two groups, one treated and the other not [16,41]. The main exclusion criteria were pregnancy [16,36,39,41], severe Graves' ophthalmopathy [36,38], chronic heart [16,36–41,43–45], liver [36,43,45] or renal [16,36,41,43,45] failure, use of any chronic treatment [36,37,39,43] or treatment influencing HRV parameters [38,40,42,44], hyperthyroid heart disease [38,44], thyroid cancer [38], diabetes mellitus [38,39,41,43,45], hypertension [38,40,41,43], cardiac arrhythmia [38,39,43,44] and smoking [41].

Population

Sample size ranged from 18 [37] to 659 [42], for a total of 495 patients with untreated hyperthyroidism, 471 with treated hyperthyroidism and 781 healthy controls.

Age was reported in all studies. The mean age was 38.4 ± 3.5 years, ranging from 31 [39] to 67 years [16].

Sex was reported in all studies except two [37,41]. The proportion of men was 18 ± 7%, ranging from 7% [36] to 38.6% [38].

Body mass index was 21.6 ± 2.6 kg/m^2 in untreated patients, ranging from 17.8[41] to 25.9 kg/m^2 [45] and 21.4 ± 0.9 in treated patients, ranging from 20.1 [41] to 26.5 kg/m^2 [16]. BMI is described in three studies before and after initiation of treatment [16,39,41] with a significant increase after antithyroid treatment (effect size = 1.48, 95%CI 0.23 to 2.73, p 0.021). BMI was not reported in five studies in untreated patients [37,40,42–44] and in eight studies in treated patients [36–38,40,42–45].

Blood pressure was 115/71 ± 5.0/4.0 mmHg in untreated patients, ranging from 109/66 [43] to 122/78 [38] mmHg and 112/69 ± 5.0/3.0 mmHg in treated patients, ranging from 107/65 [43] to 112/69 [45] mmHg. Blood pressure was not reported in eight studies in hyperthyroid patients [16,36,37,39–42,44] and in nine in treated patients [16,36–42,44].

Other characteristics were seldomly reported such as *metabolic parameters* (blood glucose levels, total cholesterol, and triglycerides) [38], *rhythm disorders* [42–45], and *smoking* [16,45].

Characteristics of hyperthyroidism

Thyroid function was described clinically in all studies, but not biologically. Four articles studied HRV parameters in untreated and treated subclinical hyperthyroidism [16,40,43,45] and seven in overt [36–39,41,42,44]. One study [37] did not report fT4 levels in untreated patients, three studies [36,37,41] fT3 levels in untreated and treated patients and two studies [37,42] TSH in untreated patients and one [42] in treated patient. Mean fT4 levels were

34.8 ± 15.0 pmol/L in untreated patients, ranging from 14.2 [43] to 122.3 [38] pmol/L, and 14.2 ± 2.1 pmol/L in treated patients, ranging from 10.4 [37] to 30.4 [41] pmol/L. Mean fT3 levels were 11.9 ± 4.9 pmol/L in untreated patients, ranging from 3.6 [40] to 30.5 [39] pmol/L, and 4.5 ± 1.0 pmol/L after treatment, ranging from 2.6 [40] to 6.0 [39] pmol/L. Mean TSH levels were 0.05 ± 0.03 mIU/L in untreated patients, ranging from undetectable [38,39,41] to 0.23 [45] mIU/L, and 1.34 ± 0.39 mIU/L after treatment, ranging from 0.02 [41] to 4.00 mIU/L [38]. Of the studies that described thyroid function before and after treatment, fT4 and fT3 levels decrease significantly (effect size = -2.73, 95%CI -3.73 to -1.73 and -3.47, -4.78 to -2.16, respectively) and TSH levels increase significantly after treatment (2.56, 1.84 to 3.27) (P <0.001).

Etiologies of hyperthyroidism were mainly Graves' disease [36,37,39,16,44,45] from 20% [45] to 100% [36,37,39] of patients, then toxic multinodular goiter [16,43,44] affecting 11% [44] to 86% [43] of patients, and toxic adenoma [16,43–45], from 4% [43] to 80% [45]. Diffuse goiter [43], hashimotoxicosis [44] and iatrogenic by levothyroxine overdose [16,40] were rarer. Others studies did not specify the etiology of hyperthyroidism [38,41,42].

Duration of hyperthyroidism was reported in 9 studies. Most studies included patients with newly diagnosed and untreated hyperthyroidism [36–39,41,44], except three that patients with untreated hyperthyroidism ≥6 months with an average of 13.7 ± 3.42 [45], or ≥12 months with an average of 12.7 [43], or ≥10 years [40]. Others studies did not indicate duration of hyperthyroidism [16,42].

Type of treatment was described in all studies. Nine studies used anti-thyroid drugs [16,36–39,41,42,44,45]: imidazols as thiamazole [36], methimazole [37], carbimazole [44] and tapazole [16], or thiouracils as propylthiouracil [44,45]. Four studies did not specify the anti-thyroid drug used [38,39,41,42]. Four studies used radioactive iodine treatment: three coupled with anti-thyroid drugs [37,42,45] and one with alone radioactive iodine treatment [43]. No study has investigated the effect of surgery. One study did not present true anti-thyroid therapy [40]. The intervention consisted of stopping Levothyroxin, introduced for the purpose of TSH-suppressive therapy, causing subclinical hyperthyroidism to return to euthyroidism. No patients were treated with beta-blockers.

Duration of treatment was 6.00 (95CI 5.99 to 6.01) months, ranging from two [41] to sixteen [44] months. Only one study did not specify the duration of anti-thyroid treatment [16].

HRV measurements and analysis

Recording of HRV measurements was ambulatory setting with normal daily activity and during spontaneous breathing. Most studies used a 24-hour holter-ECG to determine HRV [16,38,42–45], except for five studies that used a supine ECG [36,37,39–41] between five [41] and 30 minutes [39]. Almost all studies had a separate Holter monitoring system: Cambridge Electronic Design model 1401 [36,40], Life Scoope 6 [37], Marquette 3000 [38], Cardisuny 501B [39], Pathfinder 700 serial analyser [16,42], Hscribe v.3.40 [43], ECG cardioday [44], and Digital Holter Recorder [45]. Only one study did not report the monitoring system [41]. Premature atrial and ventricular beats were automatically eliminated and visually checked.

Parameters reported were both time and frequency domains in most studies, except six studies [16,40–43,45] that reported only time domain. For time domain parameters, RR intervals was reported in 9 studies [16,36–42,44], SDNN in 8 studies [16,36,38,40–42,44,45], RMSSD in 7 studies [16,38,41–45], pNN50 in 4 studies [16,38,42,44]. For frequency domain parameters, TP was reported in 4 articles [36–39], LF power in 5 studies [36–39,44], LFnu in 4 studies [37–39,44], HF power in 5 studies [36–39,44], HFnu in 4 studies [37–39,44], VLF power in 3 studies [38,39,44], and LF/HF in 5 studies [36–39,44]. All stratifications depending on severity of hyperthyroidism had more than three studies, except for RR intervals, pNN50 and frequency domain parameters in subclinical hyperthyroidism. To measure frequency domain parameters, two main methods were used: Fast Fourier transform model in three studies [36,39,44] and autoregressive model in one study [37]. One study did not specify the method used [38].
