# Supplementary material for: Effect of Hyperthyroidism Treatments on Heart Rate Variability: A Systematic Review and Meta-Analysis
Source: Biomedicines. 2022 Aug 16;10(8):1982. doi: 10.3390/biomedicines10081982 (PMC9405700; doi:10.3390/biomedicines10081982)
Supplement: Supplementary file 1 [file biomedicines-10-01982-s001.zip › BrusseauAnnexeSupplementaryOne.pdf]

Figure S1. Details for the search strategy used within each database

Pubmed
(hyperthyroidism[TW] OR hyperthyroid[TW]) **AND** ("heart rate variability"[TW] OR HRV[TW])
Filter Language = 0
Filter Dates = 0
https://pubmed.ncbi.nlm.nih.gov/?term=%28hyperthyroidism%5BTW%5D+OR+hyperthyroid%5BTW%5D%29+AND+%28%22heart+rate+variability%22%5BTW%5D+OR+HRV%5BTW%5D%29

= 35 articles

Cochrane Library
("hyperthyroidism" OR "hyperthyroid") **AND** ("heart rate variability" OR "HRV")
In All Text
Filter Language = 0
Filter Dates = 0
https://www.cochranelibrary.com/search?p_p_id=scolarissearchresultsportlet_WAR_scolarissearchresults&p_p_lifecycle=0&_scolarissearchresultsportlet_WAR_scolarissearchresults_searchType=basic&_scolarissearchresultsportlet_WAR_scolarissearchresults_searchBy=6&_scolarissearchresultsportlet_WAR_scolarissearchresults_searchText=%28%E2%80%9Chyperthyroidism%E2%80%9D+OR+%22hyperthyroid%22%29++AND++%28%22heart+rate+variability%22+OR+%22HRV%22%29

= 8 articles

Embase
('hyperthyroidism'/exp OR 'hyperthyroid'/exp) **AND** ('heart rate variability'/exp OR 'HRV'/exp)
Filter Language = 0
Filter Dates = 0

= 76 articles

Google Scholar
("hyperthyroidism" OR "hyperthyroid") **AND** ("heart rate variability" OR "HRV")
Filter Language = 0
Filter Dates = 0
In Journal articles
https://scholar.google.fr/scholar?q=(%E2%80%9Chyperthyroidism%E2%80%9D+OR+%22hyperthyroid%22)+++AND+++(%22heart+rate+variability%22+OR+%22HRV%22)&hl=fr&as_sdt=0,5&as_vis=1&as_rr=1

= 519 articles
