# Supplementary material for: Effect of Hyperthyroidism Treatments on Heart Rate Variability: A Systematic Review and Meta-Analysis
Source: Biomedicines. 2022 Aug 16;10(8):1982. doi: 10.3390/biomedicines10081982 (PMC9405700; doi:10.3390/biomedicines10081982)
Supplement: Supplementary file 1 [file biomedicines-10-01982-s001.zip › BrusseauAnnexeSupplementaryThree.pdf]

Figure S3. Quality of included studies

Methodological quality of included studies using the SIGN checklist, by study.

Cohort studies

- + Yes
- NA Not applicable
- ? Can't say
- - No
- - Low quality
- + Acceptable
- ++ High quality

	**Section 1:** Studies conception														**Section 2:** Global evaluation of the paper			
	Appropriate and clearly focused question	Comparable groups	Participation rate i.e flow chart	Performance bias	Number of dropped outs (%)	Characteristics of dropped outs	Clearly defined outcomes	Blind assessments	Compared process measures of not blind	Reliable assessment of exposure	Reliable assessment of outcomes	More than one assessment of exposure	Confusion bias	Presence of confidence intervals	Minimise the risk of bias and to establish a causal relationship	Clear evidence of an association between exposure and outcome	Results directly applicable	**General level of evidence**
Burggraaf 2001	+	-	-	-	+	-	+	-	+	+	+	-	-	+	+	+	+	+
Cacciatori 1996	+	+	-	NA	+	-	+	-	-	+	+	+	-	+	+	+	+	+
Cai 2018	+	+	-	NA	+	-	+	-	-	+	+	+	+	+	++	+	+	+
Chen 2006	+	+	-	NA	+	-	+	-	-	+	+	+	+	-	+	+	+	+
Falcone 2014	+	+	+	NA	NA	NA	+	-	-	+	+	+	+	-	+	+	+	+
Kabir 2009	+	+	-	-	NA	NA	+	-	-	+	+	+	+	+	+	+	+	+
Kaminski 2012	+	NA	NA	NA	NA	NA	+	NA	-	+	+	+	-	+	+	+	+	+
Osman 2004	+	+	+	NA	+	-	+	-	-	+	+	+	-	+	+	+	+	+
Wustmann 2008	+	NA	NA	NA	+	NA	+	NA	-	+	+	+	-	-	-	+	+	+

Randomized controlled studies

	Selection bias				Comparability bias		Outcome bias				
	Appropriate and clearly focused question	Assignement of subjects is randomised	Adequate concealment method is used	Subjects and investigators blind about treatment	Treatment and control groups are similar	Only difference between group is treatment	Standard and valid outcomes	Number of dropped outs (%)	Analyses in randomly allocated groups	Results are comparable for all sites	**General level of evidence**
Eustatia-Rutten 2008	+	+	?	+	+	+	+	+	+	NA	+
Yönem 2002	+	+	?	-	+	+	+	?	+	NA	+

SIGN	**Methodology Checklist 3: Cohort studies**		
Study identification (*Include author, title, year of publication, journal title, pages*)			
Guideline topic:		Key Question No:	Reviewer:

Before completing this checklist, consider:

1. Is the paper really a cohort study? If in doubt, check the study design algorithm available from SIGN and make sure you have the correct checklist.
2. Is the paper relevant to key question? Analyse using PICO (Patient or Population Intervention Comparison Outcome). IF NO REJECT (give reason below). IF YES complete the checklist..

Reason for rejection: 1. Paper not relevant to key question □ 2. Other reason □ (please specify):

Please note that a retrospective study (ie a database or chart study) cannot be rated higher than +.

Section 1: Internal validity		
In a well conducted cohort study:		***Does this study do it?***
1.1	The study addresses an appropriate and clearly focused question	Yes □ No □<br>Can't say □
SELECTION OF SUBJECTS		
1.2	The two groups being studied are selected from source populations that are comparable in all respects other than the factor under investigation.	Yes □ No □<br>Can't say □ Does not apply □
1.3	The study indicates how many of the people asked to take part did so, in each of the groups being studied.	Yes □ No □<br>Does not apply □

1.4	The likelihood that some eligible subjects might have the outcome at the time of enrolment is assessed and taken into account in the analysis.	Yes □ No □ Can't say □ Does not apply □
1.5	What percentage of individuals or clusters recruited into each arm of the study dropped out before the study was completed.	
1.6	Comparison is made between full participants and those lost to follow up, by exposure status.	Yes □ No □ Can't say □ Does not apply □

ASSESSMENT		
1.7	The outcomes are clearly defined.	Yes □ No □ Can't say □
1.8	The assessment of outcome is made blind to exposure status. If the study is retrospective this may not be applicable.	Yes □ No □ Can't say □ Does not apply □
1.9	Where blinding was not possible, there is some recognition that knowledge of exposure status could have influenced the assessment of outcome.	Yes □ No □ Can't say □ □
1.10	The method of assessment of exposure is reliable.	Yes □ No □ Can't say □
1.11	Evidence from other sources is used to demonstrate that the method of outcome assessment is valid and reliable.	Yes □ No □ Can't say □ Does not apply□
1.12	Exposure level or prognostic factor is assessed more than once.	Yes □ No □ Can't say □ Does not apply □
CONFOUNDING		
1.13	The main potential confounders are identified and taken into account in the design and analysis.	Yes □ No □ Can't say □
STATISTICAL ANALYSIS		
1.14	Have confidence intervals been provided?	Yes □ No □
**SECTION 2: OVERALL ASSESSMENT OF THE STUDY**		
2.1	How well was the study done to minimise the risk of bias or confounding?	High quality (++) □ Acceptable (+) □

		Unacceptable – reject 0	
2.2	Taking into account clinical considerations, your evaluation of the methodology used, and the statistical power of the study, do you think there is clear evidence of an association between exposure and outcome?	Yes ☐<br>Can't say ☐	No ☐
2.3	Are the results of this study directly applicable to the patient group targeted in this guideline?	Yes ◻	No ◻
2.4	**Notes.** Summarise the authors conclusions. Add any comments on your own assessment of the study, and the extent to which it answers your question and mention any areas of uncertainty raised above.		

SIGN	**Methodology Checklist 2: Controlled Trials**

Study identification (*Include author, title, year of publication, journal title, pages*)

Guideline topic:	Key Question No:	Reviewer:

Before completing this checklist, consider:

1. Is the paper a **randomised controlled trial** or a **controlled clinical trial**? If in doubt, check the study design algorithm available from SIGN and make sure you have the correct checklist. If it is a **controlled clinical trial** questions 1.2, 1.3, and 1.4 are not relevant, and the study cannot be rated higher than 1+
2. Is the paper relevant to key question? Analyse using PICO (Patient or Population Intervention Comparison Outcome). IF NO REJECT (give reason below). IF YES complete the checklist.

Reason for rejection: 1. Paper not relevant to key question ☐ 2. Other reason ☐ (please specify):

Section 1: Internal validity		
In a well conducted RCT study…		***Does this study do it?***
1.1	The study addresses an appropriate and clearly focused question	Yes ☐ No ☐<br>Can't say ☐
1.2	The assignment of subjects to treatment groups is randomised.	Yes ☐ No ☐<br>Can't say ☐
1.3	*An adequate concealment method is used.*	Yes ☐ No ☐<br>Can't say ☐
1.4	The design keeps subjects and investigators 'blind' about treatment allocation.	Yes ☐ No ☐<br>Can't say ☐
1.5	The treatment and control groups are similar at the start of the trial.	Yes ☐ No ☐<br>Can't say ☐
1.6	The only difference between groups is the treatment under investigation.	Yes ☐ No ☐<br>Can't say ☐
1.7	All relevant outcomes are measured in a standard, valid and reliable way.	Yes ☐ No ☐<br>Can't say ☐
1.8	What percentage of the individuals or clusters recruited into each treatment arm of the study dropped out before the study was completed?	

1.9	*All the subjects are analysed in the groups to which they were randomly allocated (often referred to as intention to treat analysis).*	Yes ☐ No ☐<br>Can't say ☐ Does not apply ☐
1.10	Where the study is carried out at more than one site, results are comparable for all sites.	Yes ☐ No ☐<br>Can't say ☐ Does not apply ☐
**SECTION 2: OVERALL ASSESSMENT OF THE STUDY**		
2.1	How well was the study done to minimise bias?<br>*Code as follows:*	High quality (++)☐<br>Acceptable (+)☐<br>Low quality (-)☐<br>Unacceptable – reject 0 ☐
2.2	Taking into account clinical considerations, your evaluation of the methodology used, and the statistical power of the study, are you certain that the overall effect is due to the study intervention?	
2.3	Are the results of this study directly applicable to the patient group targeted by this guideline?	
2.4	**Notes.** Summarise the authors' conclusions. Add any comments on your own assessment of the study, and the extent to which it answers your question and mention any areas of uncertainty raised above.	

Methodological quality of included studies using the STROBE and CONSORT checklist, by study

Studies included	STROBE score	CONSORT score
**Burgraaf, 2001**	52%	-
**Cacciatori, 1996**	61%	-
**Cai, 2018**	70%	-
**Chen, 2006**	66%	-
**Eustatia-Rutten, 2008**	-	60%
**Falcone, 2014**	61%	-
**Kabir, 2009**	56%	-
**Kaminski, 2012**	64%	-
**Osman, 2004**	47%	-
**Wustmann, 2008**	55%	-
**Yönem, 2002**	-	54%
