# Supplementary figures and images for: Effect of Hyperthyroidism Treatments on Heart Rate Variability: A Systematic Review and Meta-Analysis
Source: Biomedicines. 2022 Aug 16;10(8):1982. doi: 10.3390/biomedicines10081982 (PMC9405700; doi:10.3390/biomedicines10081982)

**Figure S2.** Methodological quality of included studies using the SIGN check

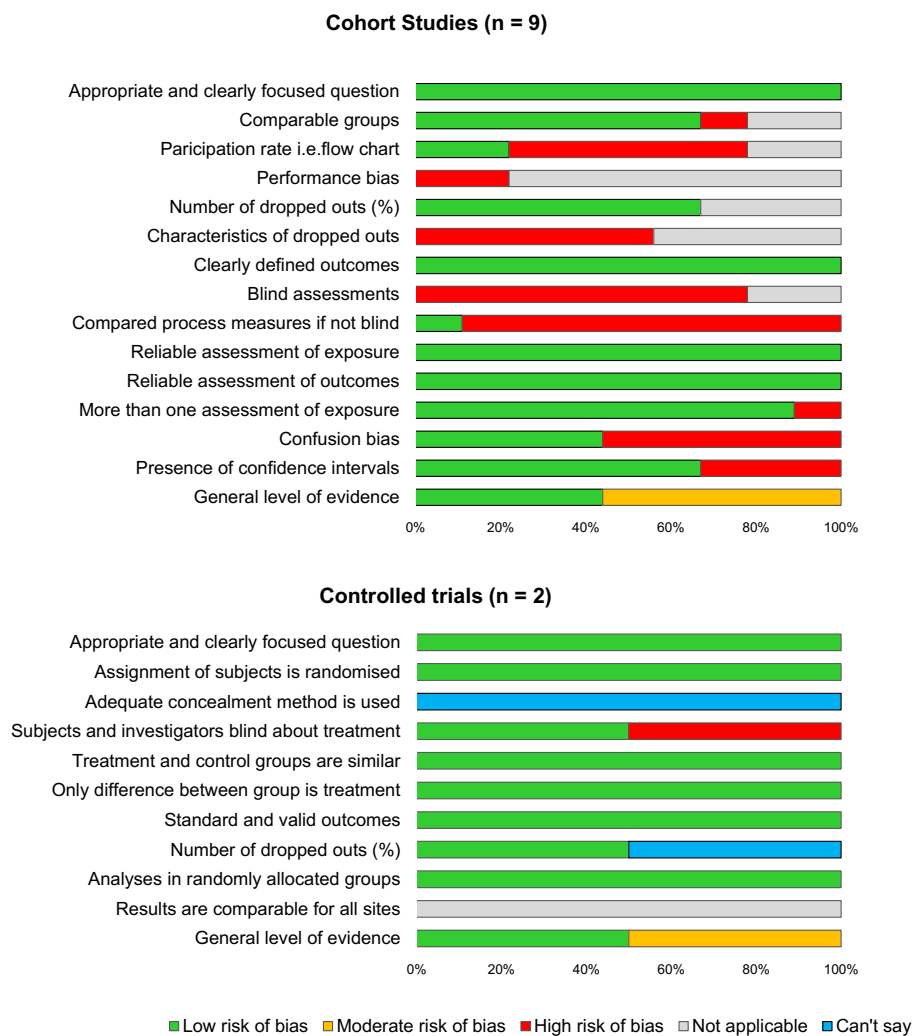

Supplement: Supplementary file 1 [file biomedicines-10-01982-s001.zip › BrusseauAnnexeSupplementaryTwo.pdf]
